# A tissue-engineered model of the atherosclerotic plaque cap: Toward understanding the role of microcalcifications in plaque rupture

**DOI:** 10.1063/5.0168087

**Published:** 2023-09-29

**Authors:** Imke Jansen, Hanneke Crielaard, Tamar Wissing, Carlijn Bouten, Frank Gijsen, Ali C. Akyildiz, Eric Farrell, Kim van der Heiden

**Affiliations:** 1 Department of Biomedical Engineering, Thorax Center Erasmus MC, University Medical Center Rotterdam, Rotterdam, The Netherlands; 2 Department of Biomedical Engineering, Eindhoven University of Technology, Eindhoven, The Netherlands; 3 Institute for Complex Molecular Systems (ICMS), Eindhoven University of Technology, Eindhoven, The Netherlands; 4 Department Biomechanical Engineering, Delft University of Technology, Delft, The Netherlands; 5 Department of Oral and Maxillofacial Surgery, Erasmus MC, University Medical Center Rotterdam, Rotterdam, The Netherlands

## Abstract

Rupture of the cap of an atherosclerotic plaque can lead to thrombotic cardiovascular events. It has been suggested, through computational models, that the presence of microcalcifications in the atherosclerotic cap can increase the risk of cap rupture. However, the experimental confirmation of this hypothesis is still lacking. In this study, we have developed a novel tissue-engineered model to mimic the atherosclerotic fibrous cap with microcalcifications and assess the impact of microcalcifications on cap mechanics. First, human carotid plaque caps were analyzed to determine the distribution, size, and density of microcalcifications in real cap tissue. Hydroxyapatite particles with features similar to real cap microcalcifications were used as microcalcification mimics. Injected clusters of hydroxyapatite particles were embedded in a fibrin gel seeded with human myofibroblasts which deposited a native-like collagenous matrix around the particles, during the 21-day culture period. Second harmonic multiphoton microscopy imaging revealed higher local collagen fiber dispersion in regions of hydroxyapatite clusters. Tissue-engineered caps with hydroxyapatite particles demonstrated lower stiffness and ultimate tensile stress than the control group samples under uniaxial tensile loading, suggesting increased rupture risk in atherosclerotic plaques with microcalcifications. This model supports previous computational findings regarding a detrimental role for microcalcifications in cap rupture risk and can further be deployed to elucidate tissue mechanics in pathologies with calcifying soft tissues.

## INTRODUCTION

Rupture of the cap of an atherosclerotic plaque can lead to cardiovascular events such as myocardial infarction and stroke.^1–3^ Cap rupture is a material failure of the tissue, which can be analyzed and predicted via stress and strain measurements. Where fibrous cap thickness was thought to be the main determinant of plaque rupture, it is now also acknowledged that the tissue composition of the cap plays a critical role.^4,5^ The atherosclerotic cap is often characterized by a heterogeneous composition and can contain inflammatory cells, apoptotic cells, and microcalcifications embedded in a collagenous matrix.^1,4–6^ According to the current definition, microcalcifications are calcified particles, no larger than 50 *μ*m,^1^ with the main component being hydroxyapatite (HA).^7–10^ Multiple mechanisms for their formation have been proposed. These include the release of extracellular vesicles,^9,11^ osteochondrogenic differentiation of smooth muscle cells,^12–14^ apoptosis,^15,16^ and a combination of these mechanisms. Microcalcifications are found to be highly prevalent throughout atherosclerotic plaques,^17^ both in the necrotic core, where they can be seen as floating debris, and in the overlying cap, the latter being possibly critical for cap rupture. Computational studies have suggested an impact of microcalcifications in the cap on mechanical stresses and concomitant rupture risk of the cap.^17–23^ Microcalcifications were shown to increase the stresses in the fibrous matrix of the cap up to twofold^17,18,24^ and to shift the maximum stress away from the shoulders of the cap to the location of the microcalcifications,^23^ suggesting a detrimental role for microcalcifications on cap mechanics and rupture. These models, however, still require experimental validation, especially, because the models assumed isotropic and homogenous mechanical behavior for the cap tissue, which is structurally heterogenous with local dominant collagen fiber orientations.^19,25,26^ Both calcification characteristics as well as collagen content contribute to plaque instability.^17^ Furthermore, processes of calcification formation and collagen organization are intertwined,^11^ showing the importance of assessing the role and interplay of both components in cap mechanics. Therefore, systematic experimental studies are needed to assess the role of microcalcifications in a collagenous matrix, to provide further insight into their effect on plaque cap mechanics.

Unfortunately, *ex vivo* material of already ruptured plaques is complex due to destroyed morphology while unruptured plaques are possibly not fully representable of the rupturing plaque. Furthermore, human *ex vivo* material has a heterogeneous composition, complicating elucidating the role of individual cap components. Alternatively, animal models are frequently used, but these are limited due to not being able to adequately mimic the human plaque mechanics. Therefore, to unravel the contribution of microcalcifications on cap mechanics, *in vitro* models are required, with variable but controllable composition, and suitable for imaging and mechanical testing. Previously we developed a tissue-engineered (TE) human fibrous cap model, where we created reproducible collagenous tissues that mimic the mechanical properties of these caps.^27^ Capitalizing on our previous studies, here we create a novel cap model with microcalcifications, mimicking analyzed human carotid plaque cap samples. We examined the effect of microcalcification inclusion on cap mechanics and found direct proof that microcalcifications lowered the ultimate tissue stress, indicating a role of microcalcifications in cap vulnerability and mechanics.

## RESULTS

### Microcalcification size and distribution in human carotid plaque caps

**FIG. 1.**
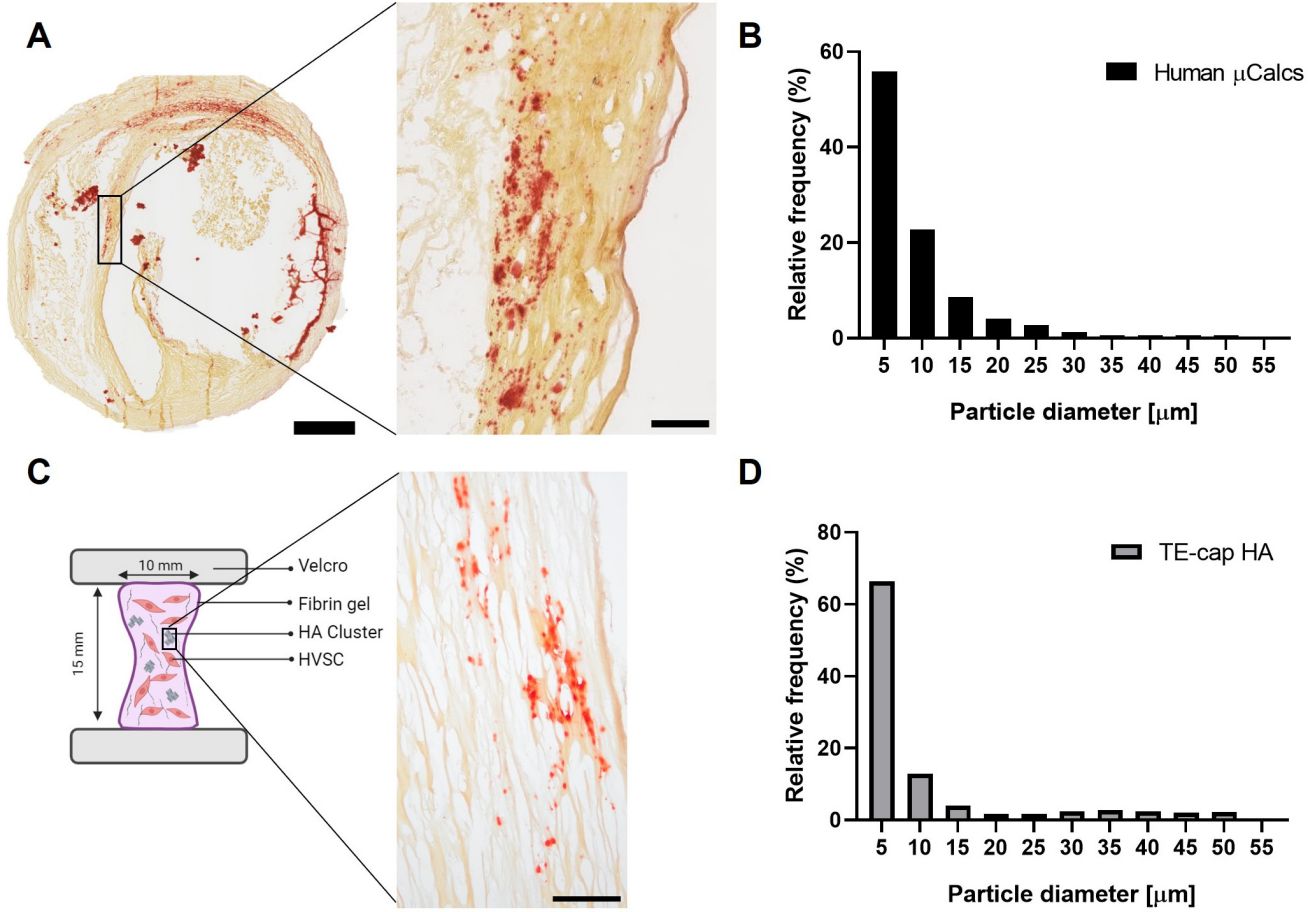
Comparison of microcalcifications in the human atherosclerotic cap to HA particles in the TE-cap. (a) A representative image of an atherosclerotic cap sample, stained with alizarin red for calcification. The zoom-in shows a cluster of microcalcifications. (b) Microcalcification size distribution of human CEA samples. (c) Graphical representation of the TE-cap with a zoom-in showing an alizarin red stained section, showing HA particles. (d) HA particle size distribution in our TE-cap. Scale bars: (a) 1 mm and 100 *μ*m, (c) 100 *μ*m.

In order to accurately model human microcalcifications, we analyzed microcalcification distribution, size, and abundance in human atherosclerotic carotid plaques [Fig. 1(a)]. Ninety tissue sections were histologically analyzed, of which 44 contained cap tissue. Microcalcifications were observed to be present in plaque cap, both in the shoulder and mid-cap regions as well as in the necrotic core and close to the media layer. In 23 sections, microcalcifications were observed in the atherosclerotic cap and analyzed, with the total microcalcification content (area fraction) within the entire cap being 5% ± 4%. Microcalcifications were distributed nonuniformly throughout the cap and in clusters [Fig. 1(a), zoom]. The clusters were of an ellipsoidal shape in the circumferential direction. In total, 1472 individual microcalcifications were identified within these clusters. Their size ranged between 2 and 52 *μ*m [Fig. 1(b)], with the majority (84% of all particles) being smaller than 15 *μ*m and only a subset (3%) being larger than 30 *μ*m. Microcalcification particle size varied between patients (mean from 3.8 ± 3.1 *μ*m for the cluster with smallest mean particles to 10.6 ± 6.8 *μ*m for biggest), shown in representative images and histograms (supplementary material Fig. 1).The region of interest was established (i.e., the area of the cluster), after which the percentage of the area taken up by microcalcification particles within this cluster was calculated (14% ± 11%).

### HA particles within TE-cap mimic microcalcifications in human cap

HA particles (n = 780) were analyzed and ranged between 1 and 55 *μ*m, with 82% being between 0 and 15 *μ*m [Figs. 1(c) and 1(d)]. Particle size between human microcalcifications and purchased HA particles were similar (p = 0.93). No abundant cell death was observed of HVSCs after the addition of HA particles compared to control (supplementary material Fig. 2), and MTT data showed no significant differences with and without HA particles (supplementary material Fig. 3). Based on the microcalcification characteristics of human cap data [Fig. 1(a)], TE-caps were created with clustered HA particles [Fig. 1(c)]. Multiple clusters were created (on average 5 per sample) and within each cluster, HA particles comprised 11% ± 6% of the area of the cluster, as analyzed with bright-field microscopy, comparable to human cap clusters of CEA specimens (p = 0.32). Cluster size and particle density were further visually compared in Alizarin Red S stained sections and showed similar size and particle distribution [Figs. 1(a) and 1(c), zoom]. Additionally, both clusters in human samples as well as in the TE-cap showed an ellipsoidal morphology, visually orienting along the fiber directionality [Figs. 1(a) and 1(c), zoom].

**FIG. 2.**
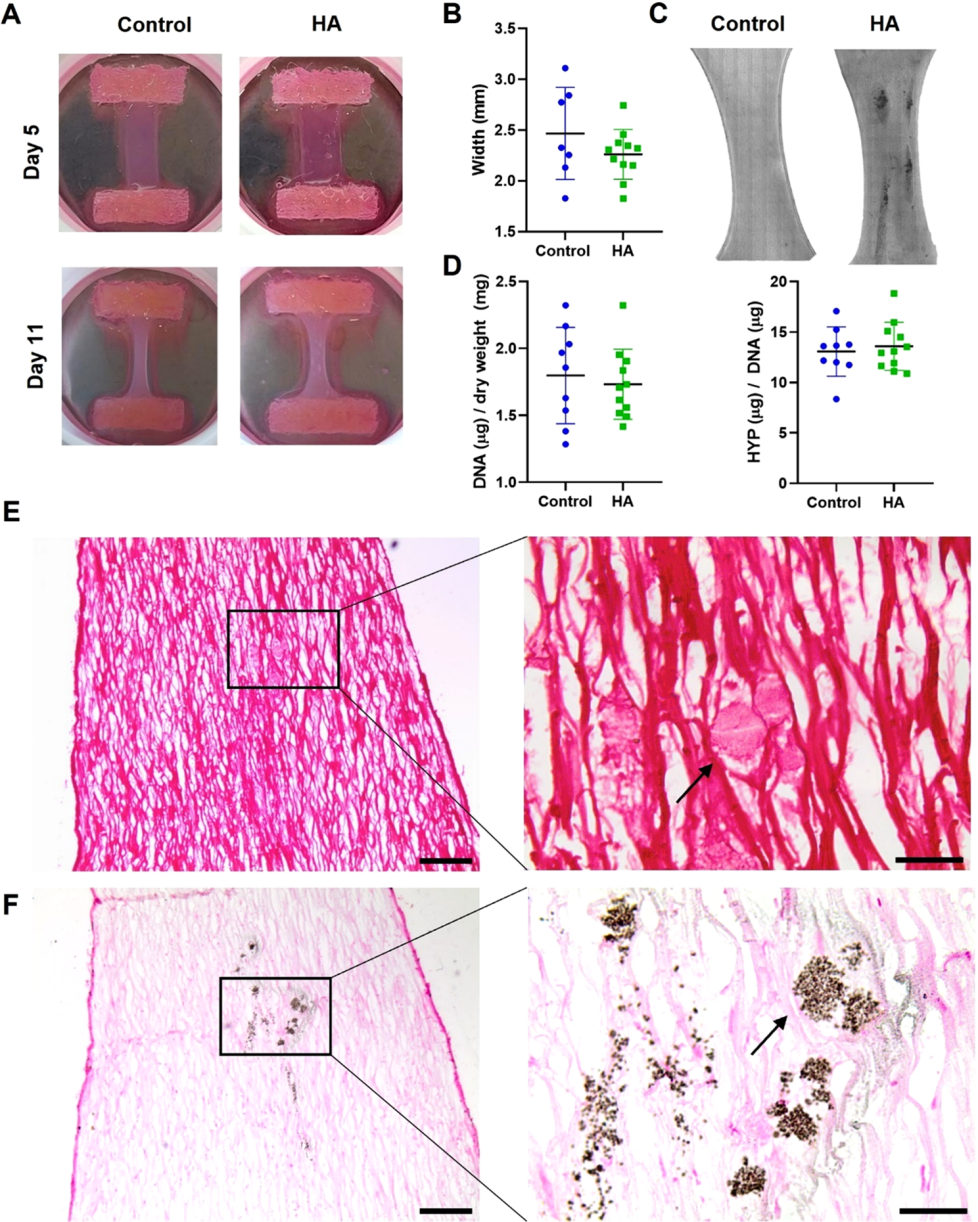
Global tissue comparison of the TE-cap models with and without clusters of microcalcifications. (a) Representative photographs showing samples at day 5 and day 21 of culture, with and without microcalcifications. (b) Compaction of two groups showing similar compaction over the culture period. (c) Tile-scan of bright-field microscopy images of a sample with HA clusters, clearly visible with this imaging method, and a control sample. (d) Cell number (DNA) and collagen (HYP) levels normalized to the total tissue mass. (e) Representative PSR staining of collagen (red/pink) with zoom-in showing HA particles located between the fibers. (f) Representative Von Kossa staining of adjacent section for HA particles (black) with Nuclear Fast Red counterstain (pink). Scale bars: (e) and (f) 200 *μ*m and zoom-in 50 *μ*m, arrow indicates same microcalcification.

**FIG. 3.**
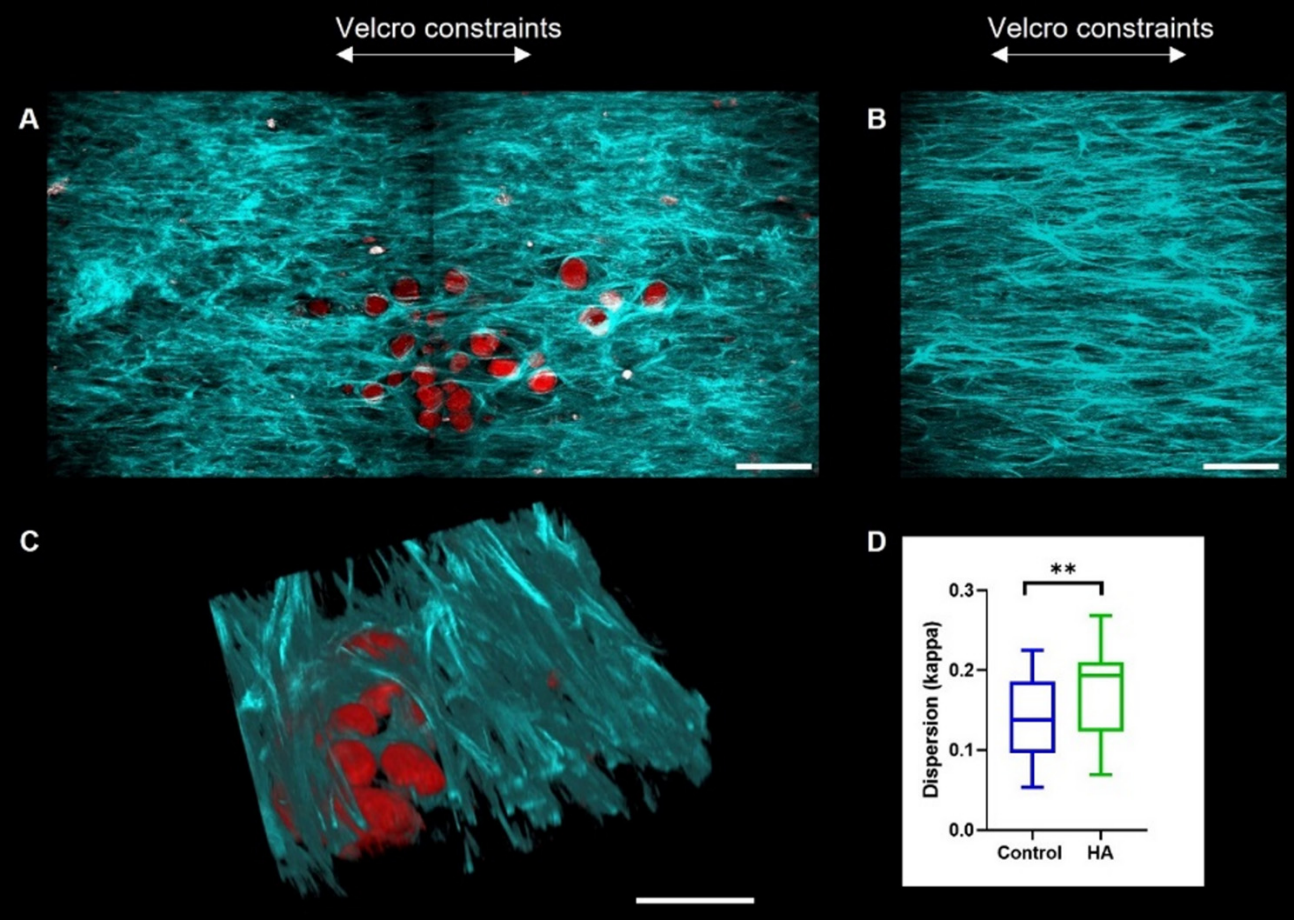
Collagen organization and microcalcification clusters at day 21. (a) Representative HA cluster visualized (red) in combination with collagen fibers (blue/green). 2 z-stacks stitched together. (b) Control area of collagen, showing collagen alignment. (c) 3D reconstruction of z-stack showing collagen matrix surrounding the HA particles. (d) κ values, a dispersion parameter, show a significantly higher dispersion for areas with HA clusters compared to control areas. Scale bars (a)–(c) 100 *μ*m.

### Composition and geometry of TE-caps

At the end of the 21-day tissue culturing period, all TE-caps showed compaction in the lateral direction [Fig. 2(a)]. No significant difference was detected between the TE-caps with and without HA [Fig. 2(b)]. Visual inspection of the bright-field images [Fig. 2(c)] demonstrated ellipsoidal shapes of HA clusters, with the major axis in the direction of the stretch applied during tissue culturing due to the Velcro constraints. They had a mean area of 0.8 ± 0.6 mm^2^ per cluster. DNA content normalized to tissue dry weight showed no differences in the amount of cells between TE-caps with and without HA [Fig. 2(d)]. Furthermore, no differences between the groups were observed in collagen produced per DNA [Fig. 2(d)].

PSR and Von Kossa stainings of adjacent sections of the TE-cap [Figs. 2(e) and 2(f)] demonstrated that the collagen fibers were aligned predominantly in the direction of the constraints and surrounded the HA particles, causing the particles to be embedded in the matrix. Visually, no differences were observed in deposited collagen matrix and cellular presence, visualized by Nuclear Fast Red staining, in proximity to the HA clusters compared to elsewhere in the same sample [Figs. 2(e) and 2(f)]. Cellular proximity to HA particles was further analyzed to rule out the effect of cellular aggregation around HA particles on local collagen deposition. No significant differences in cell number in regions with HA particles and without were found (supplementary material Fig. 4). Furthermore, HA morphology could be observed in the Von Kossa staining, revealing that the bigger HA particles (i.e., between 10 and 50 *μ*m) consisted of agglomerated microparticles [Fig. 2(f), arrow].

**FIG. 4.**
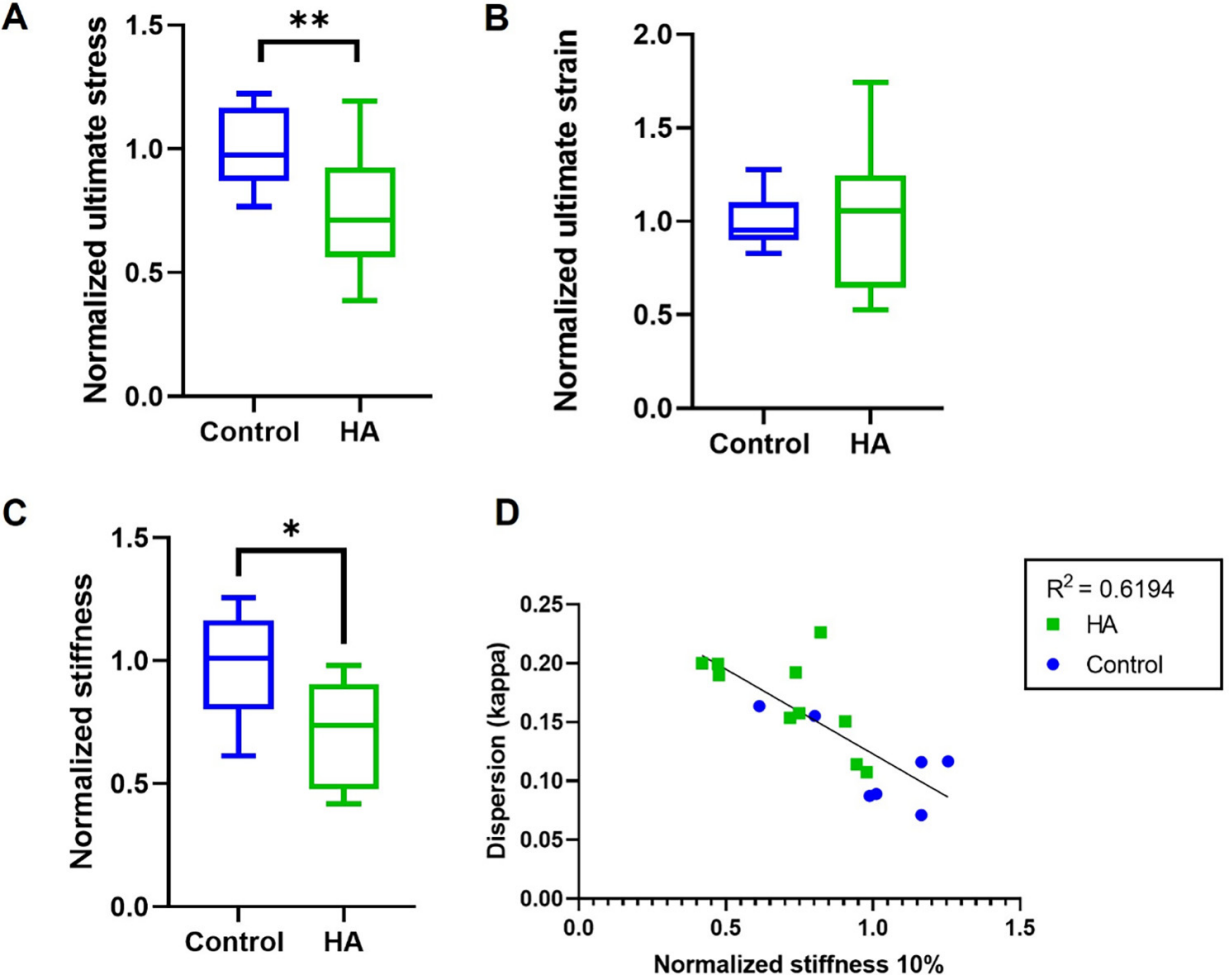
Global mechanical characterization of tissues. (a) Normalized ultimate stress showing a significant decrease for the samples with HA particles. (b) Normalized ultimate strain showing no differences between groups. (c) Normalized stiffness value at 10% strain, showing a significant decrease for groups with HA particles. (d) Correlation between the dispersion values kappa, obtained from SHG imaging and the normalized stiffness values, displaying a negative correlation.

### Effect of the presence of HA on local collagen organization

Z-stacks were obtained of 43 control areas (areas measured in samples without HA clusters) and 36 areas with HA clusters. SHG microscopy images demonstrated that HA particles were embedded in the collagen matrix, with collagen fibers bordering the particles [Fig. 3(a)]. Collagen fibers aligned in the direction of the Velcro constraints (y-direction) in control areas [Fig. 3(b)]. The 3D rendering of a cluster showed collagen fibers closely surrounding the particles [Fig. 3(c)]. For control areas the median dispersion (κ) was calculated to be 0.14 (0.01, 0.19), while for areas with HA clusters, this was 0.19 (0.12, 0.21) (p = 0.0022) (Fig. 3(d)], indicating more dispersed collagen orientation in the areas with HA clusters.

### Effect of HA on TE-caps' mechanical behavior

The ultimate tensile stress (UTS), i.e., maximum stress measured, was found to be significantly lower for samples with HA clusters (p = 0.0096) [Fig. 4(a)]. No differences were found in strain corresponding to UTS (ultimate strain) between experimental groups [Fig. 4(b)]. Samples with HA clusters were found to be significantly less stiff in the tensile direction at 10% strain (p = 0.011) [Fig. 4(c)]. These global stiffness values were correlated with the local dispersion coefficient κ, obtained from SHG imaging. A negative correlation was found between sample stiffness and collagen κ values (R^2^ = 0.691) [Fig. 4(d)].

## DISCUSSION

Within this study, we have created an *in vitro* TE model of the human atherosclerotic cap with its collagenous matrix and microcalcifications. This model can serve as an *in vitro* platform to further elucidate the role of microcalcifications on local and global atherosclerotic cap mechanics and rupture. In addition, local collagen interactions with microcalcifications can be studied, making it suitable for studying both atherosclerosis and other pathological calcification conditions. To mimic human plaque cap microcalcifications, commercially available HA particles were compared to microcalcifications of human CEA samples, after which they were incorporated in a TE human fibrous cap model. SHG confocal imaging and histological analysis were performed to assess local collagen organization in proximity to HA particles, showing that HA particles influenced the dispersion of collagen fibers. Uniaxial tensile testing unraveled the global mechanical effect of the clusters, with lowered UTS and stiffness in samples with HA clusters, revealing a role for microcalcifications on cap mechanics.

Human carotid plaque samples were analyzed for microcalcification size and density in the cap region as input to our TE model. Microcalcifications were observed in clusters, consistent with *μ*CT analysis of microcalcifications in human coronary plaques.^17^ Particle size analysis showed that the majority of the microcalcifications of human CEA plaques had a diameter between 2 and 15 *μ*m, while only a small subset (16%) was larger in size. This was also in line with previous *μ*CT analysis, where 80% was found to be between 5 and 15 *μ*m.^17^ In this study, we chose to incorporate clustering and inhomogeneous distribution in the TE model, matching with our human carotid plaque findings.

For the creation of this model, we made use of HVSCs to deposit the collagenous matrix of the TE-samples. The choice for HVSCs, a type of myofibroblast, was made because of their established role, obtained from previous experience,^27–29^ in collagen deposition and remodeling.^30^ Since adventitial myofibroblasts are furthermore thought to play a role in fibrous cap formation,^31^ the HVSCs were chosen as a suitable cell source for answering the mechanically relevant questions of this research. The fiber alignment of the collagenous matrix deposited by the HVSCs was assessed using SHG confocal imaging. In general, collagen fibers aligned along the direction of the constraints. This correlated well with the *in vivo* situation, as it was found that collagen fibers mainly align along the circumferential direction.^32^ In our TE human fibrous cap model with HA particles, the particles became embedded in the collagen matrix formed by HVSCs, as was observed both by confocal imaging and histology. Hutcheson *et al.* described that collagen can facilitate microcalcification aggregation and growth, leading to microcalcifications being formed between collagen fibers.^11,33^ Although collagen fibers formed after the addition of the HA particles in our model, the end result compared well to Hutcheson's findings and the human carotid plaque caps we analyzed histologically, with microcalcifications located in between the collagen fibers.

Our model serves as a tunable platform in which the interplay between the collagenous matrix and HA particles can be studied, and their role on tissue mechanics can be analyzed. Using our model, we detected that the incorporation of HA particles lowered the UTS, which was also observed in a recent experimental study by the group of Cardoso,^34^ where micro-beads were incorporated in a silicone-based model. The reduction in UTS could possibly be explained by a local stress increase at the tensile poles, as described in multiple computational models, which show that microcalcifications can be seen as local stress concentrators,^17,19,35,36^ an effect that could be present in our model and was also hypothesized by Cardoso.^34^ However, future research should focus on rupture path analysis to confirm the hypothesis that microcalcifications directly affect cap mechanics and rupture. Another factor to take into consideration with regard to tissue mechanics is the collagenous matrix, since this is the main load-bearing component of the fibrous cap.^32,37^ In our model, it was shown that HA particles influence the dispersion of collagen fibers and since collagen isotropy was shown to lower rupture stresses,^38^ the HA-induced effect on collagen matrix dispersion could play a role in the lowered UTS. Currently, it is yet unknown if either the dispersity of the collagen fibers or the local inhomogeneity caused by the microcalcifications is the causative mechanism of lower tissue strength. Additionally, samples with HA clusters had a lower global stiffness when compared to samples without these clusters. This may be counterintuitive, since literature on calcified plaque tissue often shows higher stiffness compared to non-calcified tissue,^1^ but these are data on macrocalcifications instead of microcalcifications. The lower stiffness of samples with microcalcifications could be attributed to the local higher collagen dispersion, as dispersed fiber orientations were found to reduce tissue stiffness when compared to highly aligned fibers,^39,40^ further highlighting not only the effect of HA inclusions but their effect on collagen architecture, thus indirectly affecting cap mechanics.

With our model, we were able to assess the effect of clustered HA particles on collagen organization and tissue strength and stiffness. However, in computational models, it has been discovered that there are various characteristics of microcalcifications that can influence the local stress accumulations they cause. It was found that they have the most consequential effect depending on their size,^34,41^ location,^24^ shape,^17,36^ and spacing.^35^ With regard to size, it has been shown that microcalcifications smaller than 5 *μ*m should not be biomechanically dangerous.^41^ Second, the location of the particles is of importance, as microcalcifications located in a thin cap compromise the mechanical stability of the cap the most.^24^ Furthermore, it has been shown that ellipsoidal particles could be even more harmful than spherical microcalcifications.^17,36^ Finally, local stress levels can increase up to a factor five if the microcalcifications are closely spaced and aligned along the tensile axis.^35^ Since these are all computational findings, future use of our model could experimentally validate or dismiss these findings by adjusting the concentration, size range, and shape of the particles used.

## CONCLUSION

In conclusion, we have created a TE fibrous cap model with a mimic of microcalcifications to study the effect of microcalcifications on atherosclerotic cap mechanics, and found a relation between microcalcification inclusions and lowered UTS and stiffness. Since cap mechanics change due to the iterative interplay between cap components and environmental cues,^29,42,43^ plaque rupture is stochastic. Many of the underlying mechanisms are still unknown, while understanding these could enhance risk assessment by identifying new imaging biomarkers and therapeutic agents. This model can serve as an *in vitro* cell-based model to further understand local interactions between collagen fibers and calcification, with regard to tissue mechanics and rupture.

## METHODS

### Characterization of microcalcifications in human carotid plaque caps

Human carotid plaque samples were obtained from seven patients that underwent carotid endarterectomy (CEA) in the Erasmus University Medical Center Rotterdam, the Netherlands. Samples were acquired in a manner that fulfilled the declaration of Helsinki and was approved by the hospital's Ethical Research Committee (MEC 2008–147). Histological cross sections of 5 *μ*m thickness (n = 90) were obtained and classified into three groups according to the American Heart Association (AHA) plaque classification. Segments with pathological intimal thickening (PIT, n = 15, 17%) showed areas of inflammatory infiltration without necrotic tissue. The fibrous cap atheroma (FCA, n = 38, 42%) consisted morphologically of a necrotic core and overlying fibrous cap with inflammatory cells. The fibrocalcific segments (FCALC, n = 37, 41%) contained heavy areas of calcification within the necrotic core. Microcalcification localization, clustering, density, and size were analyzed by Alizarin Red S staining, using FIJI Image J software (US National Institutes of Health, Bethesda, MD, USA). Particle size was calculated by creating an eight-bit image of the alizarin stained section, after which the image threshold was set, so that only alizarin red stained particles were selected. Particle measurements were performed using the build-in particle analysis tool in ImageJ.

### The effect of HA particles on human vena saphena cell (HVSC) viability

As a mechanical equivalent of microcalcifications, HA particles (Plasma Biotal Limited) were used. The effect of HA particles on HVSC viability was assessed by culturing HVSCs in a monolayer in the presence of HA particles. HVSCs were isolated from two donors (63-year-old female and 72-year-old male) after coronary bypass surgery according to previously established protocols^30^ conform the Dutch advice for secondary-use material. HVSCs were seeded in 12 well plates at a seeding density of 5000 cells/cm^2^. After 24 h, HA particles (0.05 mg/ml) were added to the culture medium and cultured for an additional 10 days. The medium was changed every 3 days, and at each medium change, bright-field images were taken to assess cell morphology. No new HA particles were added to the culture medium, as these particles attached to the cells and the wells plate and thus stayed in culture the entire period. After 10 days, a MTT (Thiazolyl Blue Tetrazolium Bromide, M5655, Sigma) assay was performed according to the manufacturer's protocols to analyze the metabolic activity of the cells.

### Creation of TE plaque caps

TE plaques without HA particles were created following the methodology described previously.^27^ To summarize, human vena saphena cells (HVSCs) (1.5 × 10^6^ cells/ml) were seeded in 1.0 × 1.5 cm-sized fibrin gels, a suspension of bovine fibrinogen (10 mg/ml, Sigma F8630), and bovine thrombin (10 U/ml, Sigma T4648), cast between two Velcro strips (1 cm long). After seeding, the samples were cultured in a growth medium consisting of advanced DMEM (ThermoFisher) supplemented with 10% Fetal Bovine Serum (Life Technologies), 1% Glutamax (Gibco), 0.1% gentamycin (Invitrogen), 1:167 Fungizone (Invitrogen), and L-ascorbic acid 2-phosphate (vitamin C, 0.25 mg/ml, Sigma A8960) for 21 days under static conditions (37 °C, 5% CO_2_). For the first 7 days of culture, ε-Amino Caproic Acid (ε-ACA, 1 mg/ml, Sigma) was added to prevent fibrin break-down.^28^

### Incorporation of HA clusters in TE plaque caps

To create tissues with HA clusters, a suspension of bovine fibrinogen and HA microparticles (0.25 mg/ml) was created. Directly after seeding of the suspension fibrinogen, thrombin, and HVSCs between the Velcro strips, clusters of 3 *μ*l HA particle-fibrin suspension were injected with a pipette, just before complete gelation of the HVSC-fibrin gel. The samples were then placed in an incubator to set for 30 min, after which medium was added. Samples were subsequently cultured for 21 days under static conditions.

### Imaging of TE plaque caps

#### Assessment of HA cluster incorporation in TE plaque caps

Bright-field microscopy images (Olympus CKX41, Olympus Life Science) of the areas with HA clusters were taken every 7 days, as well as images of the control samples without clusters. Images were captured by the Olympus SC30 camera, making use of the AnalySIS getIT software (Olympus Soft Imaging Solutions GmbH). Global tissue geometry was assessed and images were used to assess size and localization of HA clusters and particles within these clusters. The mean size of HA particles was characterized using Image J analysis.

#### Assessment of collagen organization in TE plaque caps with and without HA clusters

After the culturing period, the samples (n = 13 with HA clusters and n = 11 control) were rinsed with Phosphate Buffered Saline (PBS). The samples with HA clusters were incubated with an HA-targeting probe (IVISense Osteo 680 Fluorescent Probe, Osteosense, PerkinElmer), diluted 1:200 in PBS at 4 °C for 48 h. Following incubation, samples were rinsed with PBS and were pinned to a silicone-filled (Sylgard 184, VWR, Germany) Petri-dish with sterile surgical needles. PBS was added to fully submerge the sample. A multiphoton microscope (TCS SP5 Confocal, Leica, Germany) with a Chameleon Ultra multiphoton laser (710–1040 nm) (Coherent, USA) was used to visualize collagen architecture and HA clusters. First, a bright-field tile scan of the sample was made to localize HA clusters. Next, second harmonic generation (SHG) using two-photon microscopy (excitation of 880 nm) was employed to image collagen fibers in combination with confocal microscopy of the HA clusters (excitation of 680 nm). Z-stacks (tile size 739 × 739*μ*m, step size 3 *μ*m, pixel size 1.4 × 1.4 *μ*m) to a depth of approximately 200 *μ*m were collected in areas with and without HA clusters.

For data analysis, the maximum intensity projection (MIP) of each scanned tile was obtained and further analyzed using the Fiblab^44^ software to extract the orientation of individual collagen fibers. The predominant angle and the dispersion (κ) of the fiber orientations^45^ were measured per tile to assess the (an)isotropy of the engineered tissues.

### Tissue analysis

After SHG imaging, two samples from each group were further processed for histological examination. The samples were rinsed with PBS, fixed in 3.6% formaldehyde for 1 h, and then stored at 4 °C for later histological analysis. The other samples (n = 11 with HA and n = 9 control) were used for mechanical testing until rupture.

### Mechanical characterization: Uniaxial tensile testing

Uniaxial tensile tests were performed after SHG imaging to assess the effect of HA clusters on TE caps' mechanical properties. Before testing, samples were rinsed in PBS and imaged with a high frequency, high spatial resolution ultrasound system (VEVO 3100, FUJIFILM VisualSonics, Canada) using a linear transducer (MX550) to assess the dimensions of the central region of the sample. For uniaxial tensile testing, a custom-designed set-up^46,47^ equipped with a 20 N load cell (LCMFD-20N, Omega Engineering, USA) was used. The samples were placed, in the uniaxial tensile tester using the Velcro constraints, and the tests were performed while the samples were submerged in PBS at 37 °C. A pre-load of 0.05 N was applied to remove tissue slack and 10 cycles of preconditioning up to 10% strain^48^ were performed before the final uniaxial tensile stretching cycle until complete rupture at a strain rate of 200%/min.

Effective tensile engineering stress–strain behavior of the samples for the final uniaxial tensile stretch cycle was calculated, and ultimate tensile stress and strain, the tangential modulus at 10% strain, were assessed.^49,50^ Cross-sectional area measurements from the ultrasound scans were used for stress calculations and gauge length for the strain measurements. After mechanical testing, the ruptured samples were stored for determining the cell (DNA) and collagen (hydroxyproline (HYP)) content.

### Tissue composition: DNA and collagen content

Samples (n = 11 with HA and n = 9 control) used for DNA and HYP determination were lyophilized, weighed, and digested in a papain digestion buffer (100 mM phosphate buffer (pH = 6.5), 5 mM L-cysteine (C-1276), 5 mM ethylene-di-amine-tetra-acetic acid (EDTA, ED2SS), and 140 *μ*g/ml papain (P4762), all from Sigma-Aldrich. Each sample was mixed with 300 *μ*l of digestion buffer in a new Eppendorf tube and placed at 60 °C for 16 h for digestion. Digested samples were vortexed and centrifuged (12 000 rpm, 10 min), and the DNA content was quantified using the Qubit dsDNA BR kit (Life Technologies, Carlsbad, California, USA) and the Qubit fluorometer (Life Technologies), following the manufacturer's instructions.

To quantify HYP content, digested samples were hydrolyzed using 16M sodium hydroxide. Subsequently, HYP content was quantified using the Chloramin-T assay, including trans-4-hydroxyproline as a reference (Sigma, H5534).^48^ HYP content was then normalized for DNA content.

### Tissue composition: Histology

7 *μ*m thick cryosections, cut in the z-direction, were analyzed to evaluate global collagen matrix structure and HA clusters and to compare HA particle size and cluster density to the human CEA histological analysis. Fibrillar collagen could be visualized with Picrosirius Red (PSR), while HA particles were stained with Von Kossa and Alizarin Red S. For the PSR staining, samples were stained in PSR solution for 60 min, after which they were washed in acetic acid and dehydrated in a series of ethanol. For Alizarin Red S staining, the sections were stained in Alizarin Red S solution (pH = 4.4) for 15 min, rinsed in Milli-Q, and dehydrated in acetone and acetone–xylene (1:1). The Von Kossa staining was performed by staining sections in a silver nitrate solution for 30 min on a bright light. Afterward, they were rinsed in Milli-Q, counterstained with Nuclear Fast Red, and dehydrated. Stained sections were embedded in enthalan and imaged with bright-field microscopy (Olympus BX50).

### Statistical analysis

All data are presented as mean ± standard deviation unless stated otherwise. Statistical analyses were performed using Prism (GraphPad, La Jolla, CA, USA). A Shapiro-Wilk test was performed for normality. In the case of proven normality, an unpaired T-test was performed, and a nonparametric Mann-Whitney U test otherwise. Mechanical parameters (stress, strain, and stiffness) values were normalized to the mean of control samples within each separate experiment, to adjust for cell donor variability. Differences were considered statistically significant for p values < 0.05 (visualized as ^*^p < 0.05; ^**^p < 0.01; and ^***^p < 0.001).

## SUPPLEMENTARY MATERIAL

See the supplementary material for representative images of the Alizarin Red S stained carotid endarterectomy samples of the donors, representative bright-field images of the monolayer experiment, MTT assay data, and histological images and quantification of cellular proximity to HA particles.

## Data Availability

The data that support the findings of this study are available from the corresponding authors upon reasonable request.
